# Function and regulation of transcription factors involved in root apical meristem and stem cell maintenance

**DOI:** 10.3389/fpls.2015.00505

**Published:** 2015-07-06

**Authors:** Rebecca C. Drisch, Yvonne Stahl

**Affiliations:** Institute for Developmental Genetics, Heinrich-Heine-University, Düsseldorf, Germany

**Keywords:** stem cells, differentiation, root apical meristem, quiescent center, transcription factors

## Abstract

Plant roots are essential for overall plant development, growth, and performance by providing anchorage in the soil and uptake of nutrients and water. The primary root of higher plants derives from a group of pluripotent, mitotically active stem cells residing in the root apical meristem (RAM) which provides the basis for growth, development, and regeneration of the root. The stem cells in the *Arabidopsis thaliana* RAM are surrounding the quiescent center (QC), which consists of a group of rarely dividing cells. The QC maintains the stem cells in a non-cell-autonomous manner and prevents them from differentiation. The necessary dynamic but also tight regulation of the transition from stem cell fate to differentiation most likely requires complex regulatory mechanisms to integrate external and internal cues. Transcription factors play a central role in root development and are regulated by phytohormones, small signaling molecules, and miRNAs. In this review we give a comprehensive overview about the function and regulation of specific transcription factors controlling stem cell fate and root apical meristem maintenance and discuss the possibility of TF complex formation, subcellular translocations and cell-to-cell movement functioning as another level of regulation.

## Introduction

Terrestrial plants are sessile organisms and have to adapt to different environmental stimuli by coordinating their growth and development accordingly. Because of these needs plants have evolved a high degree of developmental and morphological plasticity, which is only possible due to the continuity of plant development (Bradshaw, 1965; Palmer et al., 2012). Plants, in contrast to animals, have to produce most of their organs post-embryonically. Therefore, plants possess structures called meristems that contain pluripotent stem cells, which are maintained during the whole lifespan of the plant. There are two main meristems in plants, the shoot apical meristem (SAM) generating above-ground tissues and organs and the root apical meristem (RAM) giving rise to the primary root of the plant. The RAM and the SAM show different structural organizations, but both meristems harbor stem cells, which continuously generate new cells (Benfey and Scheres, 2000). In *Arabidopsis*, on average four slowly dividing cells, the quiescent center (QC), maintain the adjacent stem cells (or initials) and act as a long-term reservoir for the surrounding shorter-lived stem cells (van den Berg et al., 1997). The stem cells continuously divide asymmetrically generating new stem cells still in contact with the QC cells and daughter cells, undergoing further cell divisions, are shifted further away from the QC and finally differentiate.

**FIGURE 1:**
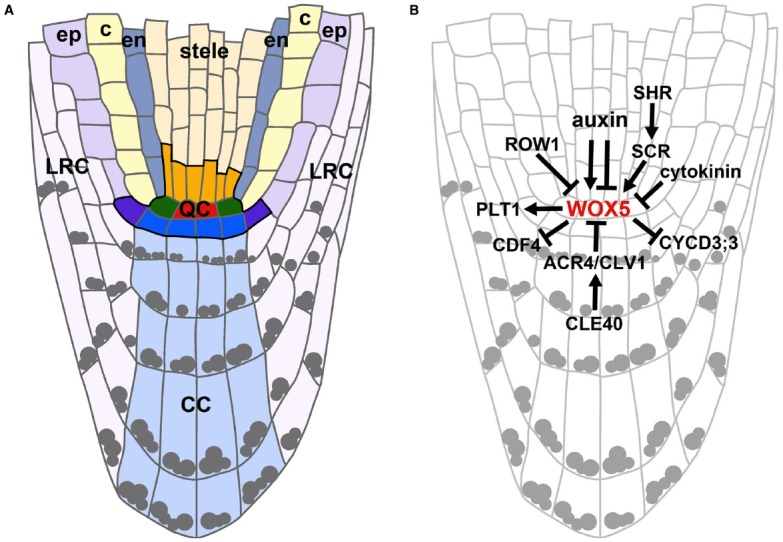
***Arabidopsis* meristematic zone organization and regulation of *WOX5* expression in the QC. (A)** Schematic representation of an *Arabidopsis* meristematic zone. The stem cell niche (outlined in black) contains the QC cells (red), the cortex/endodermis initials (green), stele initials (orange), lateral root cap/epidermis initials (purple), and CSCs (blue). ep, epidermis; c, cortex; en, endodermis; LRC, lateral root cap; CC, columella cells. Gray dots indicate starch granules. **(B)** Regulational model highlighting the complex interplay of phytohormones, TFs and signaling peptides on *WOX5* expression levels and domain. Arrows indicate positive, barred lines indicate negative regulations.

The RAM can be divided into three main zones: (a) the meristematic zone at the root tip containing the stem cell niche, (b) the elongation zone, containing the cells that after cell divisions have left the meristematic zone and are now elongating, and (c) the differentiation zone, containing cells that have acquired their destined cell fates. The beginning of the differentiation zone is marked by the appearance of root hairs (Dolan et al., 1993). The position of the stem cells remains the same throughout development and defines the cell fates of their descendants. Thereby concentrically organized clonal cell lineages are generated representing a spatio-temporal developmental gradient. From the outside to the inside of the root these cell layers are the epidermis, cortex, endodermis, pericycle, and vasculature. Cortex and endodermis together are also referred to as ground tissue. Stem cells for the lateral root cap (LRC)/epidermis and the columella are positioned distal to the QC. The columella stem cells (CSCs) give rise to the differentiated columella cells (CCs) which contain starch granules for graviperception (see Figure 1A).

Apart from phytohormones, intercellular signaling processes mediated by small peptide ligands and their respective receptors in interplay with specific transcription factors (TFs) play important roles in maintaining stem cell homeostasis in the root. In this review we will focus on the function and regulation of known TFs important for stem cell regulation in the RAM (summarized in Table 1).

**TABLE 1:** **Transcription factor (TFs) in RAM maintenance**.

**Name**	**Class/type**	**Expression domain**	**Function**	**Mobile**	**Interaction**	**Reference**
WOX5	Homeodomain	QC	Maintains surrounding stem cells; represses QC divisions	Yes		Sarkar et al. (2007), Forzani et al. (2014), Pi et al. (2015)
PLT1-4	AP2/ERF	RAM, mainly QC	Necessary and sufficient for RAM maintenance	Yes (PLT2)		Aida et al. (2004), Galinha et al. (2007)
SHR	GRAS	Stele	QC fate specification and maintenance, asymmetric cell divisions, cortex, and endodermis specification	yes	SCR SIEL JKD MGP BIB	Di Laurenzio et al. (1996), Helariutta et al. (2000), Wysocka-Diller et al. (2000), Nakajima et al. (2001), Welch et al. (2007), Long et al. (2015b)
SCR	GRAS	QC, CEI, endodermis	QC fate specification and maintenance; asymmetric cell divisions, cortex, and endodermis specification		SHR JKD MGP BIB	Scheres et al. (1995), Di Laurenzio et al. (1996), Sabatini et al. (2003), Welch et al. (2007), Long et al. (2015b)
SPT	bHLH	Epidermal initial and CSC, stele	Regulates RAM size and QC cell number		ALC IND	Groszmann et al. (2010), Girin et al. (2011), Makkena and Lamb (2013)
TMO7	bHLH	Adjacent to hypophysis	Embryonic root initiation	Yes	SIEL	Schlereth et al. (2010)
MYC2	bHLH	Ground tissue, vasculature, epidermis, LRC	JA-mediated inhibition of root growth and meristem development; repressing PLT expression			Boter et al. (2004), Chini et al. (2007), Chen et al. (2011), Fernández-Calvo et al. (2011)
UPB1	bHLH	LRC, vasculature	Modulates the balance between cell proliferation and differentiation by controlling ROS production	Yes		Tsukagoshi et al. (2010)
NTT	Zinc finger	Hypophysis and lens-shaped cell (embryo); QC, CEI, CSC, CC	Initiation of the root meristem; confers CSC fate			Crawford et al. (2015)
BRX	BRX family	Vasculature	Regulates RAM size by mediating BR and auxin signaling	Yes	NGA1	Mouchel et al. (2004, 2006a,b), Scacchi et al. (2009)
FEZ	NAC-domain	CSC and LRC/epidermis stem cells	Stimulates periclinal divisions in the LRC/epidermis initials and CSCs			Willemsen et al. (2008), Bennett et al. (2010, 2014)
SMB	NAC-domain	Maturing root cap cells	Constrains CSC-like activity and promotes differentiation; activation of root cap maturation together with BRN1&2			Willemsen et al. (2008), Bennett et al. (2010, 2014), Fendrych et al. (2014)
BRAVO	R2R3-MYB	QC, vascular initials, CEI	Repressor of QC divisions; counteracting BR signaling		BES1	Vilarrasa-Blasi et al. (2014)
BES1	BZR1-like	RAM	Activates QC divisions; represses BRAVO		BRAVO	Vilarrasa-Blasi et al. (2014)
ERF115	ERF	Dividing QC cells	Regulates QC divisions			Heyman et al. (2013)

Summary of the function, interaction, and mobility of key TFs involved in RAM development and maintenance.

## TFs Involved in RAM Development

One of the most important TF regulating stem cell fate in the root is the homeodomain containing WUSCHEL-RELATED HOMEOBOX5 (WOX5). *WOX5* is expressed in the QC in embryos and mature roots and maintains the surrounding stem cells in a largely unknown non-cell autonomous way. WOX5 is necessary for the maintenance of CSCs as in *wox5* mutant roots, cells in the CSC position acquire starch granules like differentiated CCs (Sarkar et al., 2007). Furthermore, it has been shown that WOX5 is necessary to prevent cell divisions in the QC by repressing *CYCD3;3* expression thereby establishing quiescence in the embryonic root and maintaining it in the mature root (Forzani et al., 2014; Figure 1B). WOX5 homologs have been identified in a number of plant species (Nardmann et al., 2009; Zhao et al., 2014) and were shown to be expressed in the QC in rice and maize (Kamiya et al., 2003b; Nardmann et al., 2007).

Members of the AINTEGUMENTA-LIKE (AIL) family of APETALA2/ETHYLENE RESPONSE FACTOR (AP2/ERF) domain TFs, like PLETHORA1–3 (PLT1–3) and BABYBOOM (BBM/PLT4) have been described as master regulators of root meristem initiation and maintenance. The *PLT*s are expressed in the embryonic and adult root meristem, overlapping in their expression domains mainly in and around the QC building a developmentally instructive protein gradient, where protein levels decrease the more differentiated the cells are (Aida et al., 2004; Galinha et al., 2007). *plt1 plt2* double mutants show a severe reduction in root meristem size and loss of QC markers (Aida et al., 2004). Ectopic over-expression of *PLT* leads to accumulation of stem cells in the root meristem and can also lead to the production of ectopic roots from the shoot apex (Galinha et al., 2007). The AIL genes are conserved throughout the plant kingdom and play important roles in meristem development, e.g., in adventitious rooting in poplar (Rigal et al., 2012) and gametophore stem cell formation in the moss *Physcomitrella patens* (Aoyama et al., 2012).

The GRAS-transcription factors SHORTROOT (SHR) and SCARECROW (SCR) are required for QC specification and the formative asymmetric cell divisions that are necessary for the formation of distinct cortex and endodermal cell layers (Sabatini et al., 2003). *SHR* is expressed in the stele of the *Arabidopsis* root and the protein moves one cell layer outwards into the endodermis, cortex/endodermis initial, and QC cells where it activates *SCR* expression. Loss of SHR and SCR results in a short root phenotype and abnormal QC cells indicating the importance of theses TFs in maintaining the root stem cell niche (Di Laurenzio et al., 1996; Helariutta et al., 2000; Wysocka-Diller et al., 2000; Nakajima et al., 2001). SHR/SCR act in parallel with PLT to maintain QC identity and stem cell homeostasis in the *Arabidopsis* root (Aida et al., 2004). TFs act by regulating the expression of downstream genes and some of these direct transcriptional targets have been reported. CYCD6;1 has been identified as downstream target of SHR/SCR transcriptional regulation, directly linking the asymmetric cell division in the cortex/endodermis initials with activation of cell cycle genes (Sozzani et al., 2010). Furthermore, *WOX5* expression requires SHR and SCR (Sarkar et al., 2007). The RETINOBLASTOMA-RELATED (RBR) protein has been found to physically bind to SCR and together with the cell cycle regulator CYCD6;1 and proteins of the BIRD family defines the position of the asymmetric cell divisions in the stem cell area of the root (Cruz-Ramírez et al., 2012; Long et al., 2015b). SHR and SCR regulate *CYCD6;1* expression and also expression of the BIRD family members *MAGPIE* (*MGP*) and *NUTCRACKER* (*NUT*, Levesque et al., 2006; Cui et al., 2007; Welch et al., 2007). In the monocot rice, which has a different morphology and number of cortical tissue layers, two SHR and SCR homologs each have been identified and might play a similar role in cortex and endodermis specification than in *Arabidopsis* (Kamiya et al., 2003a; Cui et al., 2007).

Other TFs have been described to play important roles in root stem cell maintenance, e.g., the R2R3-MYB transcription factor BRASSINOSTEROIDS AT VASCULAR AND ORGANIZING CENTER (BRAVO). BRAVO is acting as a cell-specific repressor of QC divisions by counteracting brassinosteroid (BR)-mediated cell divisions in QC cells (Vilarrasa-Blasi et al., 2014). Recently, the putative zinc finger TF NO TRANSMITTING TRACT (NTT) and two closely related paralogs have been described to be necessary for root meristem initiation and conferring distal stem cell fate. *nww* triple mutants fail to develop the progenitor of the QC, the lens-shaped cell, in the embryo and therefore do not develop a root. Furthermore, NTT is both necessary and sufficient to confer distal stem cell identities in the root meristem (Crawford et al., 2015). The bHLH TF UPBEAT1 (UPB1) regulates the concentration of reactive oxygen species (ROS) in the *Arabidopsis* RAM. *upb1* loss-of-function mutants show an increased RAM size and therefore UPB1 is thought to keep the balance between cell proliferation and differentiation by controlling ROS production (Tsukagoshi et al., 2010). NAC domain TFs acting in LRC development have also been identified. FEZ and SOMBRERO (SMB) antagonistically control the frequency and division plane orientation of LRC/epidermis initials and CSCs (Willemsen et al., 2008; Bennett et al., 2014). SMB, together with BEARSKIN1 and 2 (BRN1,2), is also necessary for the final differentiation steps of LRC cells and regulates programmed cell death (Bennett et al., 2010; Fendrych et al., 2014). Another bHLH TF named SPATULA (SPT) has been found to regulate root meristem size. Loss of SPT results in a larger RAM due to an increased QC size as well as supernumerary divisions in initials. SPT is thought to act independently of gibberellic acid, but might regulate auxin transport or accumulation (Makkena and Lamb, 2013).

## Phytohormonal Regulation of TFs in the RAM

Phytohormones like auxin, cytokinin, brassinosteroids, ethylene, jasmonate, and gibberellic acid play fundamental roles in specification, development, and maintenance of the RAM in *Arabidopsis*. Considerable crosstalk between different hormonal pathways is necessary for integrating external and internal cues into the dynamic developmental processes of stem cell maintenance, proliferation, and differentiation. Also transcriptional regulation is controlled by phytohormones and several TFs have been shown to be regulated by and act in concert with them.

The phytohormone auxin plays a dominant role in root initiation and development. An auxin gradient is build up by local biosynthesis and polar auxin transport in the root and has its maximum in the QC (Blilou et al., 2005; Petersson et al., 2009). The PINFORMED (PIN) auxin efflux carriers control auxin distribution and thereby regulate elongation and differentiation of root cells in a complex interplay with the PLT proteins. The expression of *PLT* TFs is auxin inducible and their expression domains are defined by local auxin accumulation (Aida et al., 2004; Blilou et al., 2005; Mähönen et al., 2014). Auxin has also been proposed to act upstream of WOX5 and PLT1 as *WOX5* expression was reported to be restricted to the QC cells by auxin via AUXIN RESPONSE FACTOR (ARF) 10 and 16 (Ding and Friml, 2010), but later WOX5 and ARF 10 and 16 were suggested to act in parallel instead (Bennett et al., 2014). Furthermore, *WOX5* expression was reported to be auxin inducible and responsible for the establishment of an auxin maximum in the root tip (Gonzali et al., 2005). The specific expression of *WOX5* in the QC is not only confined by auxin, but also by a PHD domain-containing protein, REPRESSOR OF WUSCHEL1 (ROW1), that binds tri-methylated histone H3 lysine 4 (H3K4me3) in the WOX5 promoter thereby repressing *WOX5* transcription in the more proximal cells (Zhang et al., 2015; Figure 1B). Cytokinins also play a pivotal role in root meristem balance and act antagonistically to auxin. They control the switch from meristematic to differentiated cell fates by suppressing auxin signaling and transport where cells leave the meristematic zone (transition zone). This is mediated by the AUX/IAA SHORT HYPOCOTYL2 (SHY2), which is activated by cytokinin via ARABIDOPSIS RESPONSE REGULATOR1 (ARR1), but negatively influences auxin signaling and is itself negatively regulated by auxin (Dello Ioio et al., 2007, 2008). SCR has been found to suppress cytokinin signaling via ARR1 thereby also influencing auxin accumulation in the QC (Moubayidin et al., 2013). Furthermore, cytokinins have been shown to negatively regulate *WOX5* expression possibly by modulating the auxin flux in the root and promote cell divisions in the QC (Zhang et al., 2013). The rarely dividing QC cells are thought to be less stress sensitive and protected from DNA damage and can therefore maintain their longevity. Contrariwise, the surrounding stem cells seem to be more sensitive to DNA damage. It has been proposed that the QC serves as a “safe haven” for the surrounding stem cells and if stress is occurring, driven by hormonal signals like cytokinin, BR, ethylene, and JA, the QC will divide to replenish the lost stem cells (Curtis and Hays, 2007; Fulcher and Sablowski, 2009; Cruz-Ramírez et al., 2013). Brassinosteroids (BRs) have been described to act antagonistically to auxin in *Arabidopsis* RAM maintenance (Chaiwanon and Wang, 2015). BR-mediated QC divisions are repressed by the R2R3-MYB TF BRAVO. The BR-activated TF BES1 (BRI1- EMS SUPRESSOR1) can physically interact with and repress BRAVO thereby modulating QC divisions (Vilarrasa-Blasi et al., 2014). Additionally, the TF BREVIS RADIX (BRX) has been shown to mediate a feedback between auxin and BR signaling, influencing RAM size (Mouchel et al., 2006a). Ethylene has also been shown to induce QC cell divisions (Ortega-Martínez et al., 2007). Recently, the ETHYLENE RESPONSE FACTOR115 (ERF115) TF was found to act as a rate-limiting factor of QC divisions. Here, ERF115 is positively regulated by BR, but is not involved in ethylene signaling. *ERF115* is expressed in dividing QC cells and ERF115 protein abundance is negatively regulated by proteolysis leading to a convergence of BR and ethylene signaling in the RAM (Heyman et al., 2013). Jasmonate inhibits primary root growth by reducing RAM activity and results in irregular QC divisions and CSC differentiation. This is mediated by the function of MYC2/JASMONATE INSENSITIVE1 (MYC2), a bHLH TF. MYC2 has been shown to directly bind to PLT1 and 2 promoters and to repress their transcription, thereby integrating jasmonate and auxin pathways in RAM maintenance (Chen et al., 2011).

## Regulation of TFs in the RAM by Peptides and microRNAs

Phytohormones act mostly as long-range signals, other more short-range signals mediating TF regulations include small peptides, microRNAs, and movement of TFs. Small signaling peptides are also known to regulate *Arabidopsis* root development (Delay et al., 2013) and some of them have been shown to regulate TFs involved in root stem cell homeostasis. In both shoot and root meristem maintenance CLAVATA3/EMBRYO SURROUNDING REGION (CLE) peptides are known to play important roles. In the *Arabidopsis* root, *CLE40* is expressed from differentiated columella cells and regulates CSC fate via the receptor-like kinases ARABIDOPSIS CRINKLY4 (ACR4) and CLAVATA1 (CLV1). This signaling pathway influences the expression level and positioning of *WOX5* RNA (Stahl et al., 2009, 2013). The ROOT MERISTEM GROWTH FACTOR (RGF) peptide family also known as GOLVEN (GLV) or CLE-like (CLEL) possesses a conserved 14 aa domain containing the tyrosine sulfation motif Asp-Tyr (Matsuzaki et al., 2010; Meng et al., 2012; Whitford et al., 2012). RGF1 has been demonstrated to positively regulate and define *PLT* expression and protein stability (Matsuzaki et al., 2010).

MicroRNAs (miRNAs) have been shown to generate a gradient defining vascular cell types in the root. miRNA165a and miRNA166b are transcriptionally activated by SHR in the endodermis and then move through plasmodesmata to the stele regulating the expression of the homeodomain leucine zipper (HD-ZIP) TF PHABULOSA (PHB), that determines vascular cell fates (Carlsbecker et al., 2010; Miyashima et al., 2011; Vatén et al., 2011).

## Mobile TFs in RAM Regulation

Due to their rigid cell walls, plant cells are not able to move and need to communicate with each other non-cell autonomously in order to integrate external and internal cues with development and growth. About 17–29% of TFs are predicted to move either targeted or non-targeted from cell to cell (Lee et al., 2006; Rim et al., 2011). This TF movement is proposed to occur by transit through plasmodesmata, membrane-lined channels that interconnect plant cells symplastically, and thereby propagate signaling outputs.

A prominent example of a mobile TF is SHR, which is expressed in the stele of the *Arabidopsis* root, but moves one layer further where it interacts with and activates SCR. SHR is not only a mobile TF, but it notably also alters its subcellular localization. In the stele it localizes to the nucleus and cytoplasm, whereas in the endodermis it is localized mostly in the nucleus (Nakajima et al., 2001). The cytoplasmic localization of SHR is important for its movement to the outer cell layer via plasmodesmata and is regulated by phosphorylation of a specific tyrosine residue (Gallagher et al., 2004; Vatén et al., 2011). SHR movement is dependent on microtubules and is mediated by SHORT ROOT INTERACTING EMBRYONIC LETHAL (SIEL), an endosomal protein, which needs SHR and SCR for its own expression suggesting a potential feedback for SHR regulating its own directional movement (Koizumi et al., 2011, 2012; Wu and Gallagher, 2013). Furthermore, members of the BIRD family like JACKDAW (JKD) and its close homolog BALD IBIS (BIB) constrain SHR movement by nuclear retention and complex formation (Welch et al., 2007; Long et al., 2015b).

In the *Arabidopsis* embryo, the mobile bHLH TF TARGET OF MONOPTEROS7 (TMO7) is required for embryonic root initiation and also interacts with SIEL (Schlereth et al., 2010). The TF BRX translocates from the basal plasma membrane in the vasculature to the nucleus in response to auxin (Scacchi et al., 2009). But also other TFs important for root development have been shown to be able to move, like WOX5 and PLT2 (Daum et al., 2014; Mähönen et al., 2014). Recently, it was shown that WOX5 movement from the QC to the CSCs is necessary to maintain the undifferentiated state of these cells by chromatin-mediated repression of the TF CYCLING DOF FACTOR4 (CDF4) in the CSCs (Pi et al., 2015).

The TF UPB1 has been proposed to act as a mobile non-cell-autonomous signal. It is supposed to move from its expression domain in the LRC to cells of the transition and elongation zones. Here it localizes predominantly to the nucleus and positions the location of the transition zone (Tsukagoshi et al., 2010). Nevertheless, it has not yet been completely clarified how TF movement regulates stem cell and RAM maintenance.

## Outlook and Perspectives

In the *Arabidopsis* root over 300 differentially regulated TFs have been found to be expressed, but only some have an assigned function in meristem maintenance (Birnbaum et al., 2003). TFs are regulating the expression of other genes, but information on direct targets of TFs involved in RAM regulation are scarce, except for the above mentioned examples. Regulation of the TF WOX5 alone includes phytohormones, small signaling peptides, histone modifications, and cell-to-cell movement, demonstrating the diversity of control mechanisms (Figure 1B). Although, e.g., root cap development is mainly regulated by parallel pathways, also a regulatory connection between WOX5 and SMB has been described (Bennett et al., 2014). Therefore, it is tempting to speculate that there might be complex regulatory networks involved, but that some important links have not been found yet. One could speculate that not only transcriptional regulations or TF protein stability are important but that, e.g., the interaction of TFs with other TFs or proteins create differential outputs. Also, the described subcellular translocations of some TFs might represent mechanisms to regulate TF function. Movement of TFs via plasmodesmata for short-range signaling could represent yet another level of regulation (Long et al., 2015a), but if this is directional and how it is exactly controlled remains to be elucidated.

The future challenge is to develop methods that will help to analyze and consolidate the supposed complex regulatory mechanisms. Here the rise of sequencing and bioinformatic tools together with sophisticated imaging techniques will be a prerequisite to enable the necessary modeling approaches.

### Conflict of Interest Statement

The authors declare that the research was conducted in the absence of any commercial or financial relationships that could be construed as a potential conflict of interest.
